# Endlessly mono-radial annular core photonic crystal fiber for the broadband transmission and supercontinuum generation of vortex beams

**DOI:** 10.1038/s41598-019-39527-1

**Published:** 2019-02-21

**Authors:** Manish Sharma, Prabin Pradhan, Bora Ung

**Affiliations:** École de technologie supérieure, Department of Electrical Engineering, Montreal, H3C 1K3 Canada

## Abstract

We demonstrate a new guiding regime termed endlessly mono-radial, in the proposed annular core photonic crystal fiber (AC-PCF), whereby only modes of the fundamental radial order are supported by the fiber at all input wavelengths. This attribute is of high interest for applications that require the stable and broadband guiding of mono-radial (i.e. doughnut shaped) cylindrical vector beams and vortex beams carrying orbital angular momentum. We further show that one can significantly tailor the chromatic dispersion and optical nonlinearities of the waveguide through proper optimization of the photonic crystal microstructured cladding. The analytical investigation of the remarkable modal properties of the AC-PCF is validated by full-vector simulations. As an example, we performed simulations of the nonlinear fiber propagation of short femtosecond pulses at 835 nm center wavelength and kilowatt-level peak power, which indicate that the AC-PCF represents a promising avenue to investigate the supercontinuum generation of optical vortex light. The proposed fiber design has potential applications in space-division multiplexing, optical sensing and super-resolution microscopy.

## Introduction

Orbital angular momentum (OAM) beams, aka optical vortices, and cylindrical vector beams (CVB) exhibit an annular intensity profile that possesses a zero on-axis intensity due to a phase singularity and a polarization singularity, respectively^1,2^. Optical vortex beams may carry both spin and orbital angular momenta owing to circular polarization and a helically varying phase front (described by a phase term exp(*ilθ*) in the transverse plane, where *θ* is the azimuthal coordinate and *l* refers to the topological charge), respectively. The peculiar doughnut shaped intensity distribution as well as the theoretically infinite topological states and the inhomogeneous polarization have allowed researchers to push the frontiers of optical physics and explore new modalities in super-resolution imaging^3–5^, laser material processing^6–8^, optical trapping^9,10^, sensing^11,12^ and space-division multiplexing (SDM)^13,14^, to name a few.

One well-established application of vortex beams pertains to stimulated emission depletion (STED) super-resolution microscopy where an annular beam is used together with a co-aligned Gaussian beam in order to breach the diffraction limit^15^. Another intriguing area of research relates to probing of chiral light-matter interactions with potential applications in molecular spectroscopy^16^. These applications of vortex beams would benefit from the versatility of a broadband coherent source of OAM light^17^. In this regard, researchers have explored free-space methods for the generation of broadband vortex light, including: nonlinear crystals^18,19^, nonlinear gases^20^, nanostructured metamaterials^21^ and wideband *q*-plates^22,23^. However these free-space methods still harbours some technical limitations in the continuum’s wavelength coverage or in spatial dispersion. An alternate approach pertains to supercontinuum generation in nonlinear optical fibers which could extend the spectral coverage of the ensuing optical vortex beams.

To this end, photonic crystal fibers (PCF) have demonstrated key optical properties such as *endlessly single-mode* guiding, high nonlinearities and chromatic dispersion engineering, making it an ideal medium for supercontinuum generation^24,25^. Prior research was conducted on PCF and photonic bandgap fiber designs towards SDM transmission applications^26,27^. A recent study proposed a ring core chalcogenide glass photonic crystal fiber design with promising numerical results of supercontinuum generation in the infrared^28^. A fiber based supercontinnum generation of the high-order (*l* = 8) OAM beam was also experimentally demonstrated in the near-infrared spectrum using a 3.5 m long air-core fiber^29^. Both previous studies however did not specifically address the issue of the broadband stability of vortex beams guided in the fundamental radial order, which is critical to ensure high purity of the mono-annular CVB and OAM beams at the fiber output across the widest spectral range possible.

In this work, we uncover a novel waveguiding regime in the annular core photonic crystal fiber (AC-PCF) thereby supporting the *endlessly mono-radial* (EMR) guiding regime, where the fiber enforces wavelength independent “doughnut-shaped” mono-annular guided modes. The latter property is vital as it opens the possibility to achieve the broadest and purest fiber supercontinuum vortex light supported by fiber eigenmodes of the fundamental radial order. The parameterization of the AC-PCF is presented below along with the theory that allows the optimization of its modal properties, including the special EMR guiding regime. These results are validated through full-vector finite element method (FEM) calculations (using COMSOL Multiphysics package) with perfectly matched layer boundary conditions. Further, we theoretically study optimized designs of nonlinear AC-PCF towards the supercontinuum generation in the visible-near-infrared spectrum by the numerical solution of the generalized nonlinear Schrödinger equation via the split step Fourier method.

## Description of the Annular-core Photonic Crystal Fiber Design

**Figure 1:**
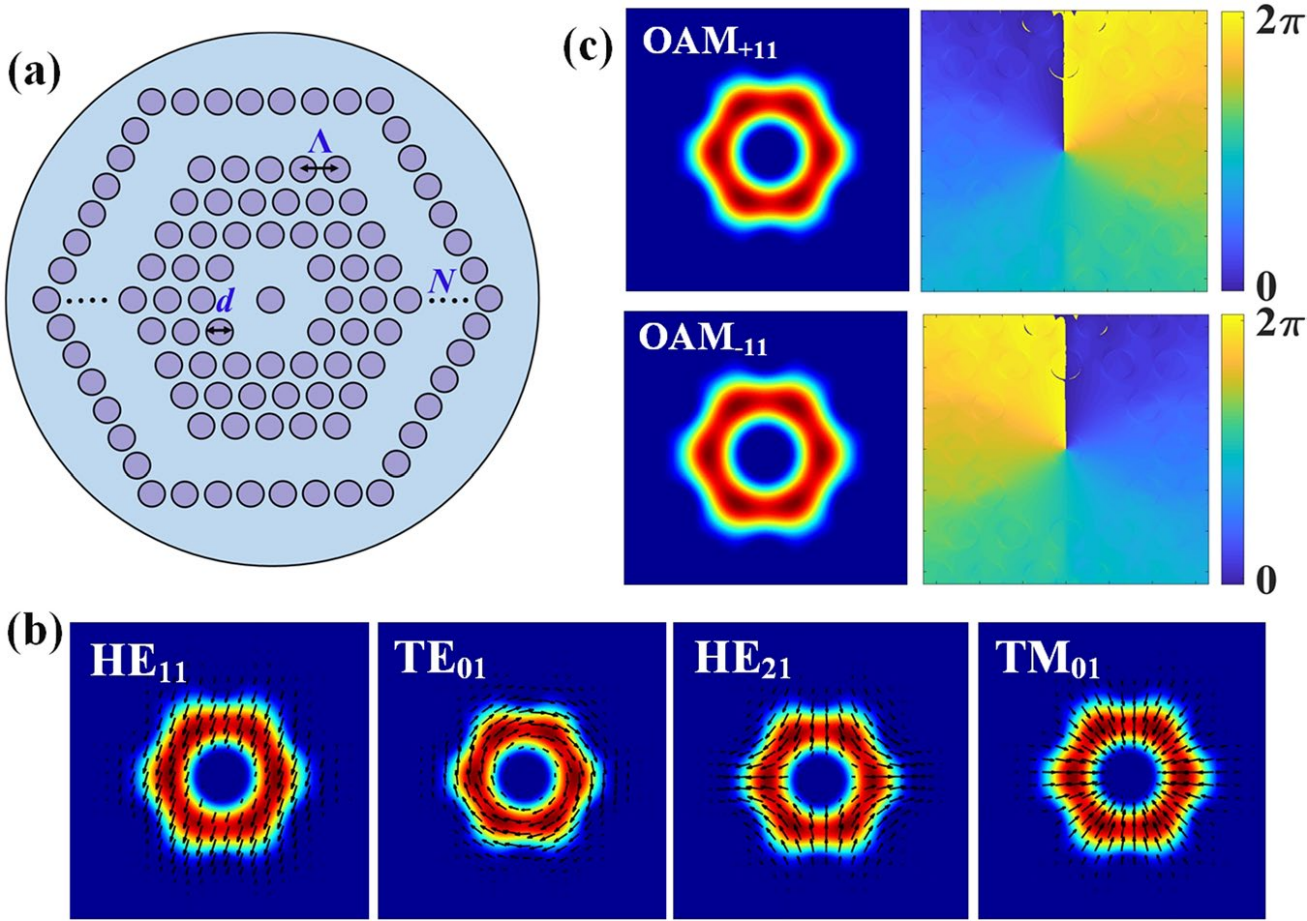
(**a**) Schematic of the cross-section of AC-PCF (**b**) Simulated intensity profiles of the first 4 guided vector modes. (**c**) Intensity and phase distributions of the OAM_±11_ modes supported by the AC-PCF.

The proposed AC-PCF structure shown in Fig. 1(a) resembles that of a standard hexagonal lattice PCF described via air hole diameter (*d*), pitch (Λ) and number of rings of air holes (*N*). The key difference though, is that in the case of the AC-PCF waveguiding occurs within a “ring” of six missing holes; while in the standard PCF the optical mode is guided around a missing center hole.

Assuming that the holey cladding can be modeled as a homogeneous material of tunable refractive index via the fill ratio *d*/Λ, one realizes that the modal properties of the AC-PCF can be investigated by means of the functionally comparable all-solid ring-core fiber^30^. Therefore the AC-PCF exhibits analogous waveguiding features such as a fundamental HE_11_ mode showing an annular intensity profile (Fig. 1(b)) along with other cylindrical vector modes TE_0*m*_, HE_2*m*_, TM_0*m*_ and other higher order mode EH_1*m*_ and HE_3*m*_ with radial order *m* = 1. In Fig. 1(c) we present the intensity and phase distributions of OAM_±11_ modes supported in a AC-PCF through the coherent superposition of hybrid modes: OAM_±11_ = HE_21(*even*)_ $\mp$ *i* HE_21(*odd*)_. We note that other higher-order OAM modes can be supported by the fiber such as: OAM_±21_ = HE_31(*even*)_ $\mp$ *i* HE_31(*odd*)_ and OAM_±21_ = EH_11(*even*)_ ± *i* EH_11(*odd*)_.

**Figure 2:**
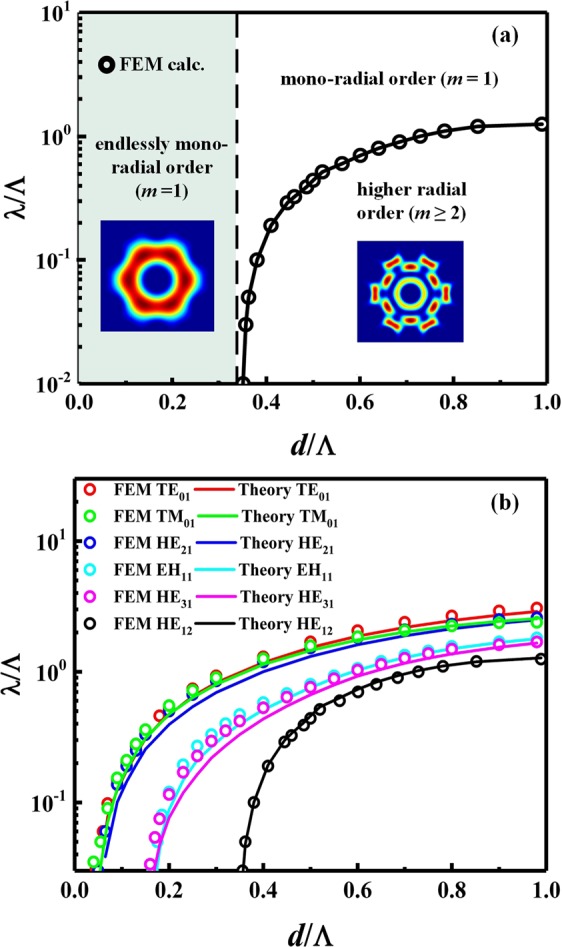
(**a**) Localization of the endlessly mono-radial-order regime in the normalized *λ*/Λ vs *d*/Λ parameter space of AC-PCFs (**b**) Modal cut-offs for the TE_01_, HE_21_, TM_01_, HE_31_, EH_11_ and HE_12_ modes predicted by analytical theory and validated by full-vector FEM.

The light guiding properties of optical fibers are commonly modelled via the dimensionless *V*-number. In the case of the AC-PCF we define this parameter as $V=(2\pi b/\lambda )(\sqrt{{n}_{core}^{2}-{n}_{clad}^{2}})$ where the effective core radius is *b* = 2Λ/$\sqrt{3}$, the core refractive index corresponds to that of the solid glass (*n*_*core*_ = *n*_*glass*_), and the refractive index of the holey cladding is defined by the effective index of the fundamental space-filling mode (*n*_*clad*_ = *n*_*FSM*_) in the triangular lattice of air-holes. Based on the latter analytical formalism, the AC-PCF enables single mode operation for *V* < 2.405, as displayed by standard optical fibers and all-solid ring-core fibers^30^. For applications previously outlined, it is crucial to prevent the onset of the first bi-annular eigenmode with *m* = 2, namely the HE_12_ mode, which occurs for *V* ≥ *V*_*cut*_; where *V*_*cut*_ = 3.832 is the HE_12_ mode cut-off. In that regard, the most prominent feature of the AC-PCF is its ability for endlessly mono-radial-order (EMR) guiding, in which case modes with fundamental radial order *m* = 1 are strictly supported by the fiber at all input wavelengths (Fig. 2(a)). The threshold of this special waveguiding regime was identified through FEM calculations for relative hole diameters less than 0.35 (namely *d*/Λ < 0.35). The ability to enforce modes with *m* = 1 inside an EMR-guiding AC-PCF helps to mitigate issues related to mode coupling with undesired higher-radial-order modes (*m* ≥ 2). The latter feature is also desirable, among others, in SDM applications using CVB and OAM beams where mux/demux operations generally assume the coaxial alignment of modes in the fundamental radial order^13^.

The full list of possible mono-radial (*m* = 1) modes supported in the EMR regime are: HE_11_, TE_01_, HE_21_, TM_01_, EH_11_ and HE_31_. Figure 2(b) demonstrates that the cut-off conditions for these modes of interest along with that of the HE_12_ mode, as predicted by the analytical model^30^, are in good quantitative agreement with the full-vector FEM numerical calculations. We recall that the fundamental HE_11_ mode has no cut-off. Small deviations between the analytical description and FEM simulations of the AC-PCF can be attributed to the fact that ring-core fibers and AC-PCF fibers are not identical in shape due to the inhomogeneous photonic crystal cladding present in the latter type of fiber, and also because the ring-core fiber model assumes the same refractive index in the inner and outer claddings (while this condition is not exactly met in the case of the AC-PCF). The procedure used for the determination of the modal cut-offs is detailed in the Methods section.

## Optimization of Fiber Parameters for Supercontinuum Generation in the Visible-Near-Infrared

**Figure 3:**
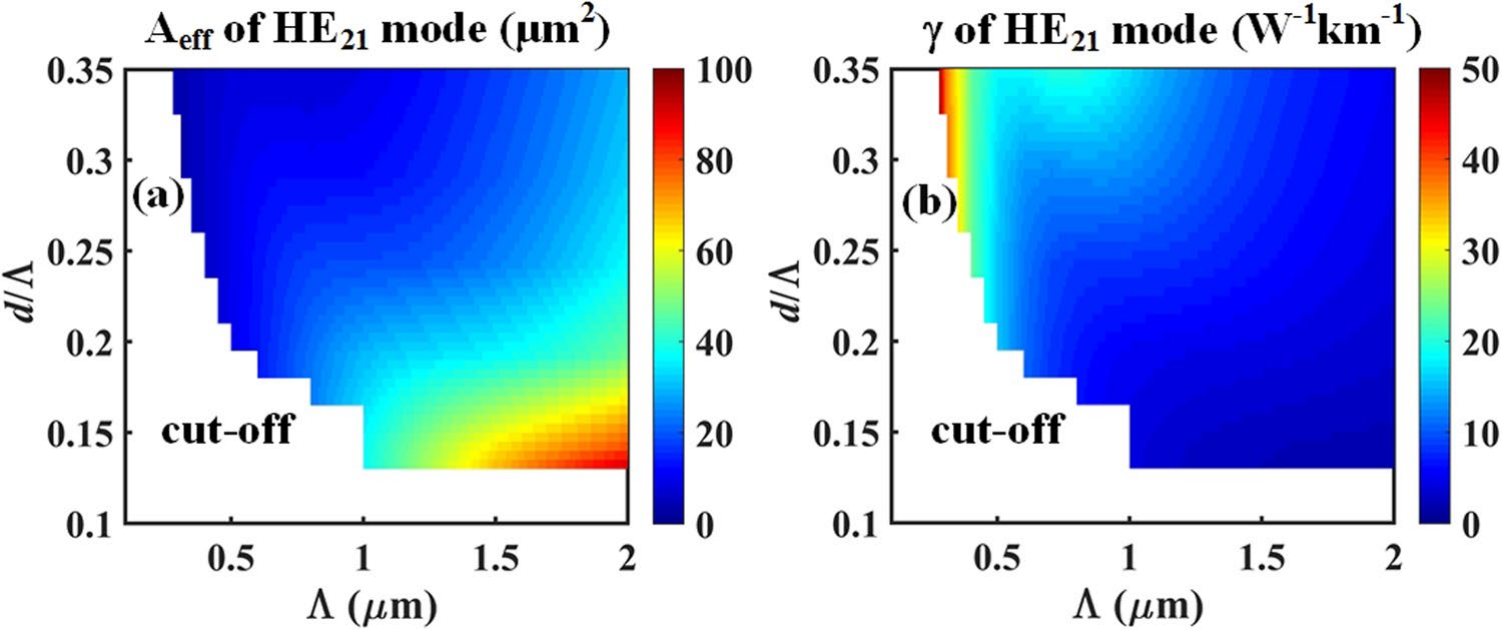
(**a**) Effective mode area, and (**b**) nonlinear parameter of the HE_21_ mode at *λ* = 835 nm in a silica AC-PCF operating in the endlessly mono-radial-order guiding regime.

**Figure 4:**
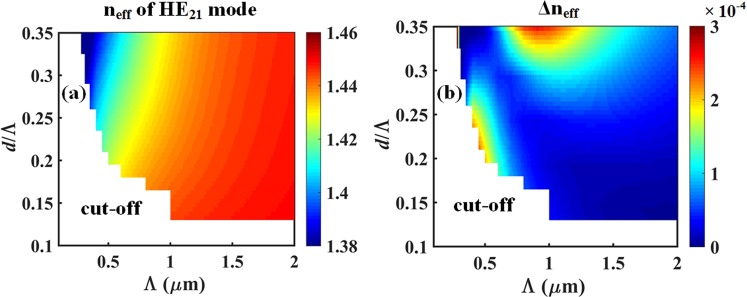
(**a**) Effective index of the HE_21_ mode and (**b**) its minimum effective index separation inside the LP_11_ group at *λ* = 835 nm in a silica AC-PCF operating in the endlessly mono-radial-order regime.

**Figure 5:**
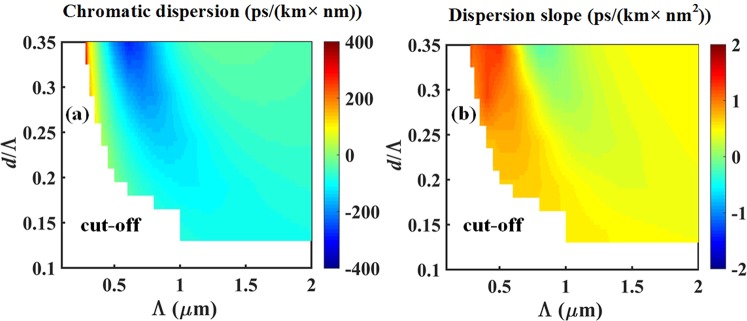
(**a**) Plot of the group velocity chromatic dispersion and (**b**) dispersion slope of HE_21_ mode at *λ* = 835 nm *in silica* AC-PCF within the endlessly mono-radial-order regime.

In this section, a fused silica glass AC-PCF is optimized for parameters relevant to supercontinuum generation (SCG) in fiber by means of guided CVB and OAM modes: high optical nonlinearity, high modal effective index separation and near-zero chromatic dispersion at the input pump wavelength of 835 nm. Figure 3 shows the value of the effective mode area (*A*_*eff*_) and nonlinear parameter (*γ*) of a silica AC-PCF operating inside the EMR waveguiding regime (*d*/Λ < 0.35) at *λ* = 835 nm for the HE_21_ mode. We note that in this section we have limited our discussion to the HE_21_ mode for concision since its modal properties very closely mirror that of the TE_01_ and TM_01_ modes, and also because the even and odd HE_21_ modes serve as the basis set to create the OAM_±11_ modes of interest. In our numerical analysis we have defined the threshold for modal cut-off as the level where less than 40% of the optical power is guided within the core region of the fiber (a detailed description of this criterion for modal cut-off is presented in the Methods section). Inexistent data corresponding to regions of modal cut-offs have duly been identified in Figs 3 to 5.

One can observe in Fig. 3(a) that the mode confinement, i.e. value of *A*_*eff*_, decreases with increase in the *d*/Λ ratio, and increases with the hole pitch (Λ). Since the nonlinear parameter is defined as *γ* = 2*πn*_2_/(*λA*_*eff*_), where *n*_2_ is the nonlinear refractive index of the glass, we observe the reverse relationship for *γ* as a function of *d*/Λ and Λ. Hence the optimization of optical nonlinearities (i.e. magnitude of *γ*) can be achieved with fiber structures presenting aspect ratios near the edge of the EMR regime (*d*/Λ = 0.35) and for smaller hole periods. However, lowering the value of Λ eventually creates a very small core region that forces the guided mode to have a significant fraction of its power leaking into the cladding region and to eventually cut-off. Figure 3(b) displays the nonlinear parameter (*γ*) values for silica AC-PCF in the EMR at *λ* = 835 nm for the HE_21_ mode. We note that the value of *γ* increases with the *d*/Λ ratio, while decreases with Λ as expected from the inverse relationship with the effective mode area. In particular, high nonlinearities can be achieved for normalized hole diameters in the 0.2 < *d*/Λ < 0.35 range and for sub-micron periods (Λ < 1 *μm*).

Moreover, the fiber is designed for the stable propagation of mono-radial OAM beams, which depends on lifting the modal degeneracy of the constituent hybrid HE/EH cylindrical vector modes^28,31,32^. A common rule of thumb is to maintain a minimum intermodal separation of the effective indices of Δ*n*_*eff*_ ≥ 10^−4^ between adjacent vector modes so as to promote their fiber transmission stability^14,32^. Figure 4(a,b) shows the value of the HE_21_ effective mode index (*n*_*eff*_) and its minimum intermodal separation (Δ*n*_*eff*_) with respect to the adjacent vector modes TE_01_ and TM_01_. Figure 4(a) indicates that the value of *n*_*eff*_ increases almost linearly with the pitch Λ, ostensibly because a larger core radius (*b* = 2Λ/$\sqrt{3}$) promotes a better *E*-field overlap within the high-refractive-index annular ring.

Figure 4(b) indicates that a parametric space with sufficient intermodal separation is found in the region roughly bounded by (0.3 ≤ *d*/Λ ≤ 0.35) and (0.8 ≤ Λ ≤ 1.6 *μ*m). Similarly, another interesting region for the stable propagation of the HE_21_ mode can be found for small fiber geometries with (0.18 ≤ *d*/Λ ≤ 0.25) and (0.3 ≤ Λ ≤ 0.7 *μ*m), although this second region is located near the modal cut-off.

## Chromatic Dispersion Engineering

The chromatic dispersion plays an important role in supercontinuum generation as it determines the extent to which spectral components of a short pulse will travel with different velocities. The undesirable effect of group velocity chromatic dispersion (GVD) is the temporal broadening of the optical pulse that ultimately affects the phase-matching and excitation of optical nonlinearities in the fiber. The GVD in the fiber is commonly described by the dispersion parameter (*D*) in units of ps/(km-nm):

1 $$D=\frac{-\lambda }{c}\,\frac{{d}^{2}{n}_{eff}}{d{\lambda }^{2}}$$

where *n*_*eff*_ is the wavelength-dependent effective refractive index of the propagating mode of interest in the single-material fiber (silica in this case). We note that the material contribution to the total chromatic dispersion was here taken into account by implementing the Sellmeier equation of pure fused silica in our simulations. Similar to what was achieved with regular PCFs^33,34^, we demonstrate below the ability to engineer the chromatic dispersion of CVBs and OAM modes in an AC-PCF through the precise tuning of its microstructure.

Henceforth, we investigated the dependence of the GVD on the geometrical parameters (*d* and Λ) of a silica AC-PCF optimized for supercontinuum generation at 835 nm input wavelength in the few femtoseconds regime. Our calculations in Fig. 5 indicate that low dispersion can be achieved inside a relatively large parameter space: (0.3 ≤ *d*/Λ ≤ 0.35) and (1 ≤ Λ ≤ 2 *μ*m). Similarly, a smaller region of low GVD occurs near modal cut-off for (0.18 ≤ *d*/Λ ≤ 0.25) and (0.3 ≤ Λ ≤ 0.7 *μ*m). Within these interesting regions of low absolute dispersion, inspection of Fig. 5(b) allows to further mitigate the effects of third-order dispersion by plotting the dispersion slope, which should also be minimized in order to enhance phase-matching with optical nonlinearities. In that regard we observe low dispersion slope values for fiber geometries with (0.3 ≤ *d*/Λ ≤ 0.35) and (0.8 ≤ Λ ≤ 1.2 *μ*m) that is co-located with a desirable region of low absolute GVD.

**Figure 6:**
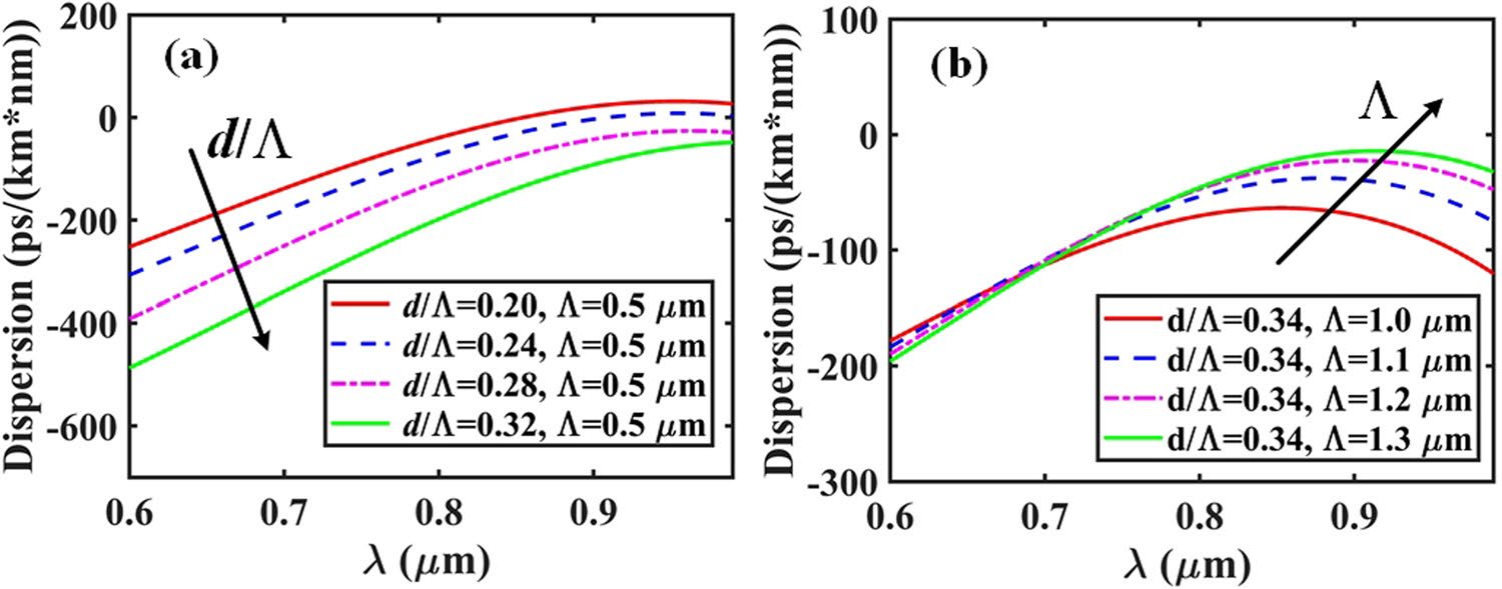
Group velocity dispersion curves for (**a**) different values of normalized hole diameters *d*/Λ with fixed period Λ = 0.5 *μ*m, and (**b**) for various periods Λ with fixed ratio *d*/Λ = 0.34.

Figure 6 plots the chromatic dispersion as a function of wavelength for a number of potentially interesting AC-PCF configurations. In particular, for a given fixed pitch value of Λ = 0.5 *μ*m, Fig. 6(a) indicates that it is possible to go from a near-zero dispersion at *λ* = 835 nm to high negative dispersion (in the normal dispersion regime), by increasing the normalized hole diameter (*d*/Λ) from 0.20 to 0.32. On the other hand if one keeps the normalized hole diameter constant while increasing the pitch, Fig. 6(b) indicates that the GVD progressively shifts toward higher positive values in the direction of anomalous dispersion.

Based on the analysis of Fig. 6, we selected two promising designs of AC-PCF (also keeping in consideration practical fiber fabrication limitations) named “Fiber 1” and “Fiber 2” for the supercontinuum generation with short optical pulses centered at *λ* = 835 nm. The structural parameters of the two optimized fiber designs and their principal modal properties are presented in Table 1. The *β* values in Table 1 denote the coefficients of the Taylor series expansion of the wavenumber *β*(*ω*) about the pulse’s center frequency (*ω*_0_)^33^. Here we note that *β*_1_ (i.e. group velocity) was omitted since the calculations of the nonlinear pulse propagation in the next Section were performed in a moving time reference frame. In addition, we confirmed that the mode purity of the generated OAM_±11_ beams using both fiber designs remained very high (>96%) across the whole simulated range of wavelengths from 600 to 1200 nm. Details of the OAM mode purity calculations based on a modal field decomposition into spiral harmonics are provided in the Methods section.

**Table 1:** Linear and nonlinear optical properties of the HE_21_ mode in two designs of silica AC-PCF optimized for supercontinuum generation at 835 nm wavelength.

	*d*/Λ	Λ (*μm*)	*γ* (*W*^−1^ *km*^−1^)	*β*_2_ (*s*^2^/*m*) (10^−27^)	*β*_3_ (*s*^3^/*m*) (10^−41^)	*β*_4_ (*s*^4^/*m*) (10^−55^)	*β*_5_ (*s*^5^/*m*) (10^−70^)	*β*_6_ (*s*^6^/*m*) (10^−84^)	*β*_7_ (*s*^7^/*m*) (10^−98^)
Fiber 1	0.34	1.3	10.6	11.25	3.21	1.89	9.89	−5.63	15
Fiber 2	0.20	0.5	14.7	4.61	7.73	1.04	26.7	−84.5	9.25

## Numerical Simulation of Supercontinuum Generation in AC-PCF

The nonlinear pulse propagation in the fiber can be simulated by solving the generalized nonlinear Schrödinger equation (GNLSE)^33,34^:

2 $$\frac{\partial A}{\partial z}+\frac{\alpha }{2}A-{\Sigma }_{n\mathrm{=2}}^{\infty }\frac{{i}^{n+1}}{n!}{\beta }_{n}\frac{{\partial }^{n}A}{\partial {T}^{n}}=i\gamma (1+i{\tau }_{shock}\frac{\partial }{\partial T})(A(z,t){\int }_{-\infty }^{t}R(t^{\prime} )\times |A(z,t-t^{\prime} ){|}^{2}dt^{\prime} )$$

where, *A* = *A*(*z*, *t*) is the electric field envelop, *α* is the attenuation constant, *β*_*n*_ is the *n*^*th*^ order dispersion coefficient [see Table 1] about the center frequency *ω*_0_, and *γ* is the nonlinear parameter given by:

3 $$\gamma =\frac{2\pi {n}_{2}}{\lambda {A}_{eff}}$$

In Eq. (3), *n*_2_ represents the nonlinear refractive index of fused silica, *λ* denotes the wavelength and *A*_*eff*_ is the effective mode area as defined by^35^:

4 $${A}_{eff}=\frac{|\int ({\overrightarrow{e}}_{v}\times {\overrightarrow{h}}_{v}^{\ast })\cdot \overrightarrow{z}dA{|}^{2}}{\int |({\overrightarrow{e}}_{v}\times {\overrightarrow{h}}_{v}^{\ast })\cdot \overrightarrow{z}{|}^{2}dA}$$

In Eq. (4), ${\overrightarrow{e}}_{v}(x,y,\omega )$ and ${\overrightarrow{h}}_{v}(x,y,\omega )$ respectively denote the transverse electric and magnetic vector field distributions which were here calculated via FEM simulations. The time derivative term on the right hand side of Eq. (2) includes the effect of dispersion owing to nonlinearity, associated with the phenomena of self-steepening and optical shock. The first term in the definition of *τ*_*shock*_ below is the dominant contribution while the second term includes the effect of the frequency-dependent effective mode area and nonlinear refractive index, as^36^:

5 $${\tau }_{shock}=\frac{1}{{\omega }_{0}}+\frac{d}{d\omega }[ln(\frac{{n}_{2}(\omega )}{{A}_{eff}(\omega )})]$$

This equation can be rewritten as:

6 $${\tau }_{shock}=\frac{1}{{\omega }_{0}}+[\frac{1}{{n}_{2}(\omega )}{(\frac{d{n}_{2}(\omega )}{d\omega })}_{{\omega }_{0}}-\frac{1}{{A}_{eff}(\omega )}{(\frac{d{A}_{eff}(\omega )}{d\omega })}_{{\omega }_{0}}]$$

In this study we have utilized Eq. (6) to determine the shock time scale that takes into account the dependence of the optical shock time on the effective mode area, while we neglected the contribution from the frequency dependence of the nonlinear index *n*_2_, since this contribution is negligibly small. The obtained values for *τ*_*shock*_ at the pump’s central wavelength (835 nm) are thus found to be 0.62 fs and 0.59 fs for Fiber 1 and Fiber 2, respectively. The total nonlinear response *R*(*t*) of the material, which includes both the instantaneous electronic and delayed ionic Raman contributions, is described as:

7 $$R(t)=(1-{f}_{R})\delta (t)+{f}_{R}{h}_{R}(t)$$

where in the case of silica glass we have *f*_*R*_ = 0.18 for the fractional contribution to the delayed Raman response, *δ*(*t*) is the Dirac function and *h*_*R*_ is the Raman response function which can be analytically modeled as:

8 $${h}_{R}(t)=\mathrm{(1}-{f}_{b})\,({\tau }_{1}^{2}+{\tau }_{2}^{2}){\tau }_{1}exp(\frac{-t}{{\tau }_{2}})sin(\frac{t}{{\tau }_{1}})+{f}_{b}[\frac{\mathrm{(2}{\tau }_{b}-t)}{{\tau }_{b}^{2}}]exp(\frac{-\,t}{{\tau }_{b}})$$

where for silica^37^ we have: *τ*_1_ = 12.2 fs, *τ*_2_ = 32 fs, *f*_*b*_ = 0.21, *τ*_*b*_ = 96 fs.

In general the GNLSE must be solved through numerical methods. To that end, the split-step Fourier method is based on parsing the linear dispersive and nonlinear contributions of the GNLSE and solving them independently over small half discretization steps^38,39^ Based on the latter approach to solving Eq. (2), we performed simulations of supercontinuum generation in a 20 cm long silica AC-PCF using the optimized parameters of Fiber 1 and Fiber 2 presented in Table 1.

**Figure 7:**
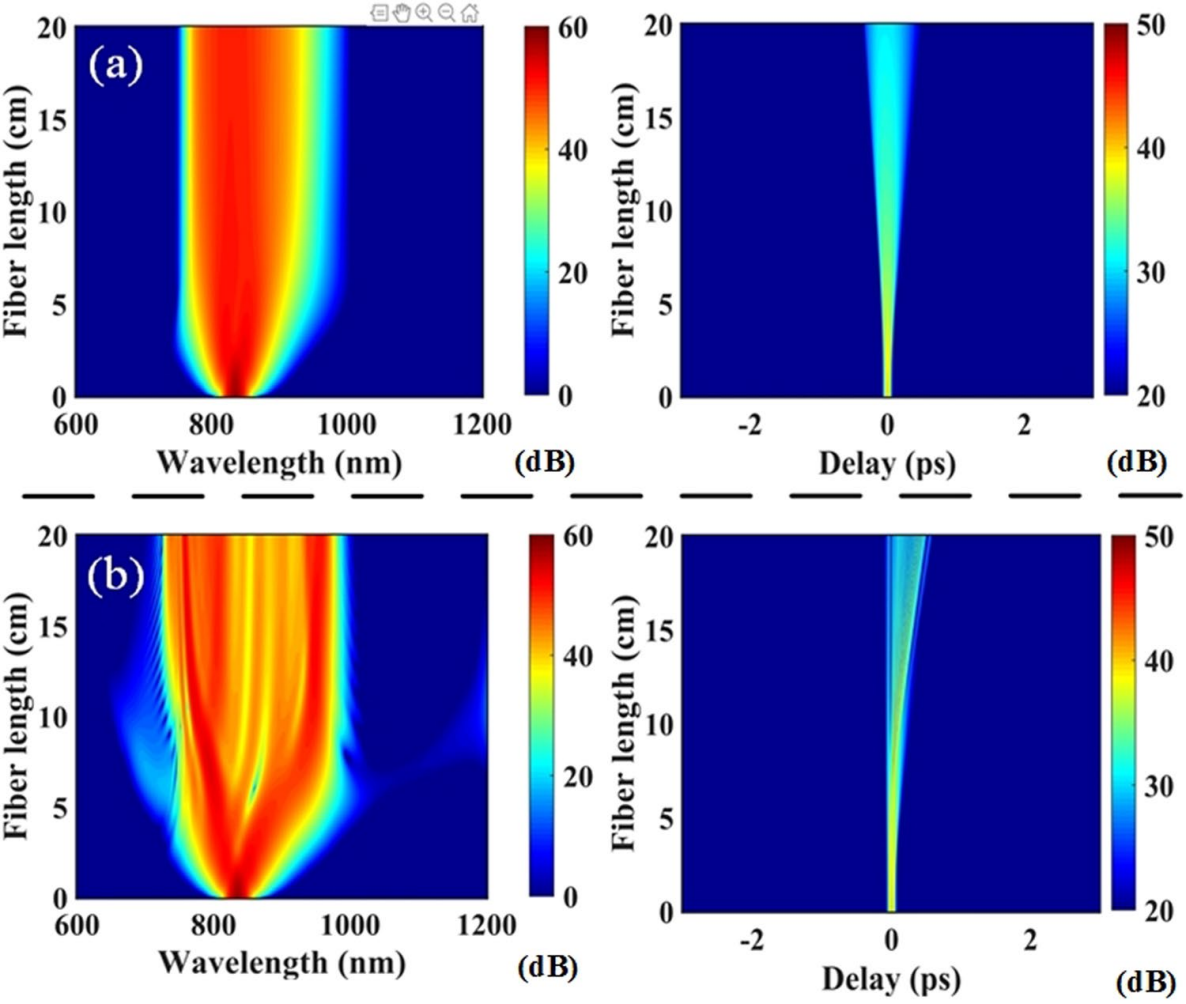
(**a**) Pulse spectral and temporal profiles as a function of propagation in (**a**) Fiber 1 with Λ = 1.3 *μm* and *d*/Λ = 0.34, and (**b**) Fiber 2 with Λ = 0.5 *μm* and *d*/Λ = 0.2 for 10 kW peak input power.

Simulations were performed using an unchirped Gaussian pulse of *τ*_*FWHM*_ = 60 fs duration and 10 kW peak power (*P*_*p*_). At *λ* = 835 nm center wavelength, Fiber 1 and Fiber 2 are both pumped in the normal dispersion regime (*D* = −30.1 and −12.56 ps/(km · nm), respectively) with nonlinear coefficient *γ* = 10.6 and 14.7 W^−1^ km^−1^. We note that since the designed fiber has no form birefringence, the effect of polarization coupling were not considered in the solution to GNLSE. Figure 7(a) shows the spectral and temporal evolution of the input Gaussian pulse in Fiber 1. Initial evolution of the spectrum is characterized by self-phase modulation and normal dispersion as these phenomena together leads to substantial spectral and temporal broadening as well as a rapid decrease in peak power over a short distance as expected^33^. The significant amount of normal dispersion in Fiber 1 limits the nonlinear spectral broadening of the input pulse, the full extent of which occurs within 3 centimeters propagation length, and is followed by the formation of additional red-shifted components due to Raman scattering. Figure 7(b) also illustrates temporal and spectral broadening of the input pulse for Fiber 2 since it is similarly pumped in the normal dispersion regime. However, the pumping occurs closer to the zero dispersion wavelength and with a larger nonlinear coefficient. The latter conditions allow for a more rapid spectral broadening and significant power transfer towards the anomalous dispersion region. In this case, we thus observe complex soliton fission dynamics coupled with Raman scattering that results in dispersive-wave generation and a fine structuring of the output pulse spectrum.

**Figure 8:**
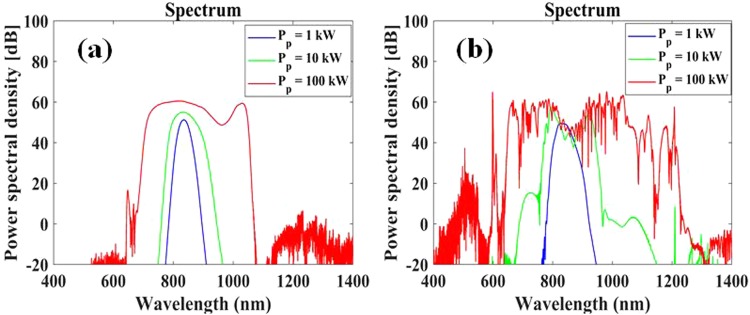
Output pulse spectra after 20 cm propagation in AC-PCF with (**a**) Λ = 1.3 *μm* and *d*/Λ = 0.34 (Fiber 1), and (**b**) Λ = 0.5 *μm* and *d*/Λ = 0.2 (Fiber 2) as a function of input pulse peak powers *P*_*p*_.

Another set of calculations was performed to simulate the output spectrum after 20 cm propagation in either Fiber 1 or Fiber 2, for various levels of peak power (1, 10, 100 kW) of a 60 fs input pulse. We note that experimental demonstrations of SCG in small-core nonlinear fibers using comparable kW-level femtosecond pulses have been demonstrated in the past^25^. Figure 8(a) shows the corresponding spectral profile simulated for Fiber 1 where we observe that nonlinear spectral broadening dominates over the effects of chromatic dispersion such that an output spectrum spanning from 696 to 1058 nm at −20 dB from the top is obtained for 100 kW peak power. In the case of Fiber 2 in Fig. 8(b), the highest peak power is shown to translate into more power towards the anomalous dispersion regime such that the mechanisms of soliton fission and dispersive wave generation are more prominently displayed and lead to a spectral broadening spanning from 644 to 1219 nm at *P*_*p*_ = 100 kW.

Hence, Fiber 2 with structural parameters Λ = 0.5 *μm* and *d*/Λ = 0.2 represents the most promising fiber design for supercontinuum generation due to its higher optical nonlinearities and lower group velocity dispersion. However, fabrication of this design with very small holes of diameter *d* = 0.1 *μm* could pose practical problems, in which case the design of Fiber 1 will represent a viable alternate solution. The proposed fiber design is amenable to fabrication via the established stack-and-draw technique. For applications in the near-to-far infrared spectrum (including the C-band) for which hole diameters are relatively large (*d* ≥ 1 *μm*) we expect no particular fabrication issues. However for applications that include the visible range (as in the present study) the holey cladding can become deeply sub-micron in size, such that particular care must be taken throughout the fiber fabrication process starting from the preform creation to the fiber drawing under precisely controlled gas pressure. Tolerance of the fiber design to imperfections in the fabrication process can be inferred from the parametric study of the modal properties shown in Figs 3–5. We remark that because the point of operation in Fiber 2 is close to cut-off, this specific design has low tolerance to perturbations in the nominal values of the structural parameters Λ and *d*/Λ. In the case of Fiber 1, Fig. 4(b) indicates that the minimum modal separation (Δ*n*_*eff*_) remains above the 10^−4^ threshold even for changes in the structural parameters as large as 10%. Figures 3 and 5 similarly indicate that Fiber 1 retains a fair degree of tolerance to structural perturbations (as large as 5%) with respect to optimal optical nonlinearities and GVD.

## Conclusion

In summary, we have studied the design and simulation of the triangular-lattice annular core photonic crystal fiber (AC-PCF) enabling both the stable broadband guided-transmission and supercontinuum generation of optical vortex beams in fiber. The analytical investigation, supported by numerical simulations, shows that a novel waveguiding regime is possible in the AC-PCF in which the fiber strictly supports modes of the fundamental radial order at all wavelengths. This special regime, here called *endlessly mono-radial*, occurs when the photonic crystal structure obeys the condition *d*/Λ < 0.35. Similarly to standard PCFs, we also show that the AC-PCF can be tailored to enhance the optical nonlinerarities as well as engineer the chromatic dispersion of the fiber. The unique waveguiding features of the proposed design thus make it an ideal medium to study mono-annular beams, namely cylindrical vector beams and orbital angular momentum beams, within a linear or nonlinear broadband regime solely limited by the transparency window of the host material. In particular, we demonstrate through numerical solutions of the generalized nonlinear Schrödinger equation that properly optimized designs of the endlessly mono-radial AC-PCF can support the supercontinuum generation of stable optical vortex beams. The work is relevant to research in the topical areas of space-division multiplexing, super-resolution microscopy and optical sensing via structured light.

## Methods

We here describe the method of validation via FEM-based calculations of the AC-PCF’s modal cutoffs with the reported full-vector analytical theory detailed in^30^ for the functionally equivalent ring-core fiber (in an effective-index model). Here we note that in the design of AC-PCF in Fig. 1(a) the first ring of air holes are missing, whilst in a standard PCF it is the central hole that is missing. A proposed definition for the effective core radius (b) of an AC-PCF is *b* = 2Λ/$\sqrt{3}$, thus exactly twice the value proposed for the PCF in^40^. The AC-PCF is further defined by its core refractive index *n*_*core*_, (i.e. refractive index of the host material) and the effective refractive index of the cladding as defined by the fundamental space-filling mode (*n*_*FSM*_) in the triangular lattice of air holes of diameter *d* and period Λ.

**Figure 9:**
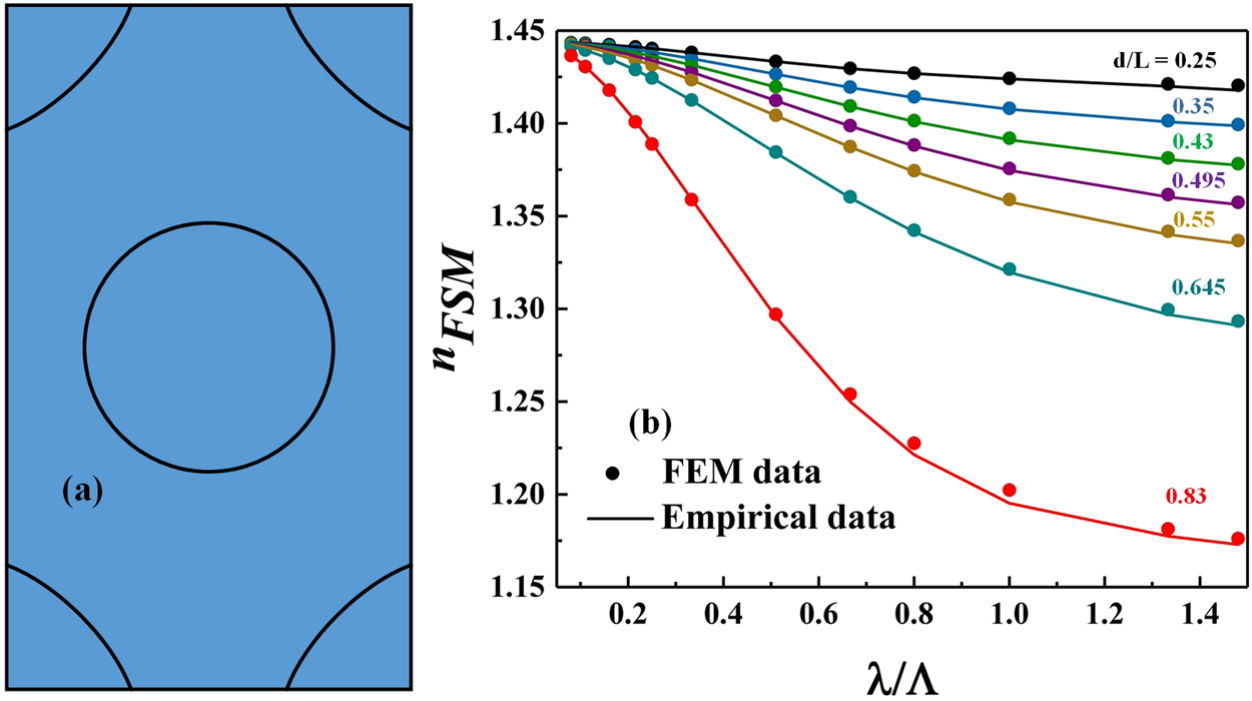
(**a**) Unit cell used in the FEM solver of the (**b**) effective index of the fundamental space-filling mode (*n*_*FSM*_) as a function of normalized wavelength (*λ*/Λ) for different values of *d*/Λ aspect ratios. A comparison between the full-vector calculations with the approximate empirical relation in^40^ is also presented.

In this work, we obtained the effective index of the fundamental space-filling mode (Fig. 9(b)) by means of the full-vector FEM eigenmode solution of the infinite triangular lattice of air holes (where periodic boundary conditions were implemented on all sides of the unit cell in Fig. 9(a)). For validation purposes, the numerical results were compared with a reported empirical model in^40^ as depicted in Fig. 9(b).

The condition of cut-off frequency for different eigenmodes (TE_0*m*_, TM_0*m*_, HE_*v*,*m*_ and EH_*v*,*m*_) of the ring core fiber are analytically defined in Eqs 9–12, respectively^30^:

9 $${J}_{0}({V}_{0}){N}_{2}(\rho {V}_{0})-{N}_{0}({V}_{0}){J}_{2}(\rho {V}_{0})=0$$

10 $${J}_{0}({V}_{0}){N}_{2}(\rho {V}_{0})-{N}_{0}({V}_{0}){J}_{2}(\rho {V}_{0})=\frac{\mathrm{(1}-{n}_{0}^{2})}{{n}_{0}^{2}}[{J}_{0}({V}_{0}){N}_{0}(\rho {V}_{0})-{J}_{0}(\rho {V}_{0}){N}_{0}({V}_{0})]$$

11 $${J}_{v-2}({V}_{0}){N}_{v}(\rho {V}_{0})-{N}_{v-2}({V}_{0}){J}_{v}(\rho {V}_{0})=\frac{\mathrm{(1}-{n}_{0}^{2})}{\mathrm{(1}+{n}_{0}^{2})}[{J}_{v}({V}_{0}){N}_{v}(\rho {V}_{0})-{J}_{v}(\rho {V}_{0}){N}_{v}({V}_{0})]$$

12 $${J}_{v+2}({V}_{0}){N}_{v}(\rho {V}_{0})-{N}_{v+2}({V}_{0}){J}_{v}(\rho {V}_{0})=\frac{\mathrm{(1}-{n}_{0}^{2})}{\mathrm{(1}+{n}_{0}^{2})}[{J}_{v}({V}_{0}){N}_{v}(\rho {V}_{0})-{J}_{v}(\rho {V}_{0}){N}_{v}({V}_{0})]$$

here, *J*_*v*_ and *N*_*v*_ are Bessel functions of the first and second kind, *v* is the azimuthal order, *m* is the radial order, *V*_0_ is the cut-off normalized frequency with

$${{\rm{V}}}_{0}={k}_{0}b(\sqrt{{n}_{core}^{2}-{n}_{clad}^{2}}),$$

where $\rho =b/a\mathrm{=(2}{\rm{\Lambda }}/\sqrt{3})/(d\mathrm{/2)}$, *n*_0_ = *n*_*core*_/*n*_*FSM*_ and the inner cladding radius *a* = *d*/2. Of particular interest is the numerical solution of Eq. (11) for the first higher-radial order HE _12_ mode which provides the cut-off condition for mono-radial order guiding.

The numerical solution of Eqs (9–12) using the above analytical formalism are compared with the cut-off frequencies obtained from the exact FEM simulation in Fig. 2(b). In the FEM simulations, the cut-off condition of all the modes is quantitatively defined based on the optical power fraction (*f*_*p*_) guided within the transverse region of interest (i.e. fiber core) as given by.

13 $${f}_{p}=\frac{|{[\int ({S}_{z})dA]}_{core}|}{|{[\int ({S}_{z})dA]}_{total}|}$$

where *S*_*z*_ denotes the Poynting vector and the core area is bounded by (0 ≤ *r* ≤ *b*). Through an iterative study, a power fraction threshold of *f*_*p*_ = 40% within the core area was identified as the criterion for modal cut-off.

**Figure 10:**
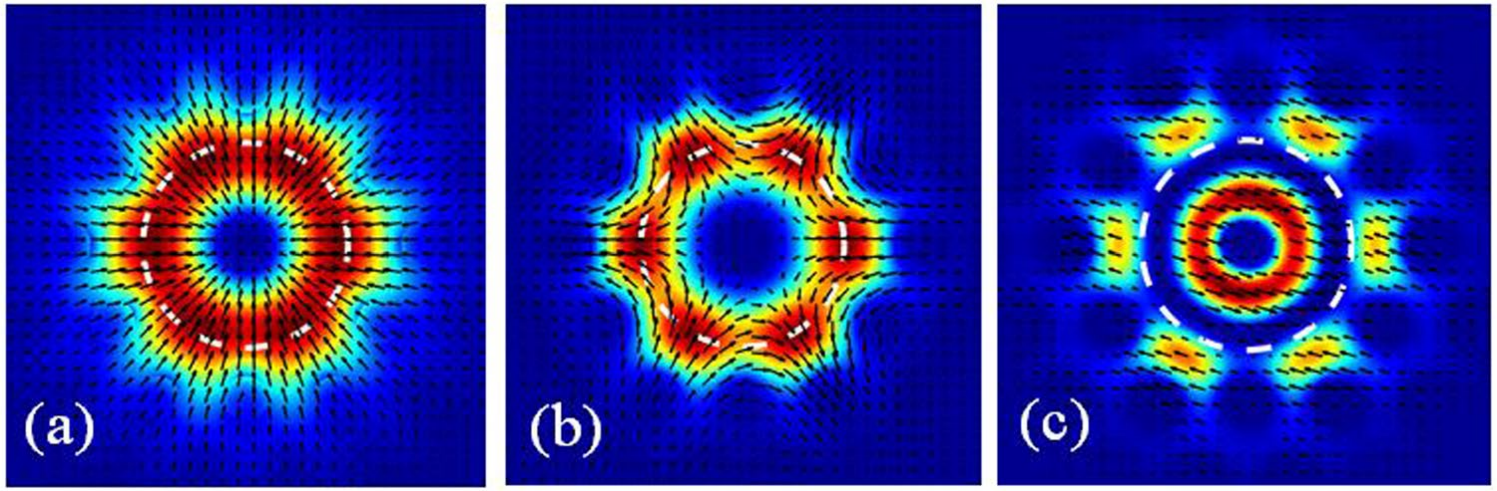
Profiles of (**a**) TM_01_, (**b**) HE_31_ and (**c**) HE_12_ modes exactly at the modal cut-off threshold when *f*_*p*_ = 40% for an exemplar AC-PCF with *d*/Λ = 0.5 where the dashed white lines indicate the core region of interest of effective radius *b* = 2Λ/$\sqrt{3}$.

Therefore all physical modes numerically found with *f*_*p*_ > 40% are considered as core-guided in our analysis; while those modes whose power fraction is below threshold are systematically rejected (i.e. cut-off). Figure 10 shows exemplar profiles of eigenmodes supported by an AC-PCF that are exactly at cut-off (*f*_*p*_ = 40%).

We have evaluated the modal purity of the OAM_±11_ beams generated in the Fiber 1 and Fiber 2 designs optimized for supercontinuum generation. To do so we performed the projection of the transverse field distribution *u*(*ρ*, *θ*) onto the “spiral harmonics” $exp(-\,i\ell \theta )$ where the variable $\ell$ denote any $\ell$-th order OAM harmonic integer. We subsequently computed the energy ${C}_{\ell }$ transmitted within each $\ell$-th OAM harmonic via^26,41^:

14 $${C}_{\ell }=\frac{1}{2\pi }{\int }_{0}^{\infty }\,|{\int }_{0}^{2\pi }\,u(\rho ,\theta )exp(-\,i\ell \theta )d\theta {|}^{2}\rho d\rho $$

Finally, the normalized power weight (${P}_{\ell }$) for each $\ell$-th OAM harmonic (i.e. topological charge number) contained in the modal field under test is written as:

15 $${P}_{\ell }=\frac{{C}_{\ell }}{{\Sigma }_{n=-\infty }^{\infty }\,{C}_{n}}$$

**Figure 11:**
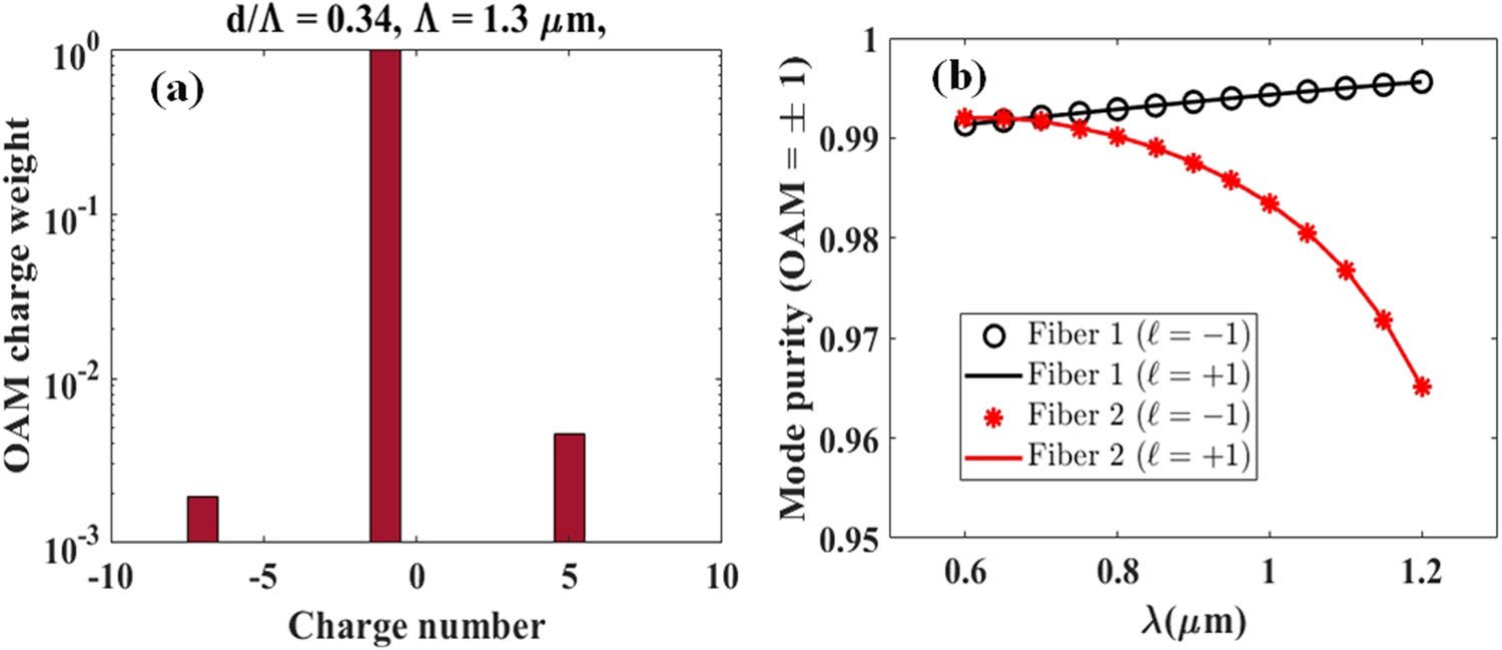
(**a**) OAM charge weights in Fiber 1 of generated OAM beam with topological charge ($\ell =-\,1$) at 835 nm (**b**) OAM mode purity as a function of wavelength for Fiber 1 and Fiber 2.

Figure 11(a) shows a typical “OAM spectrum” obtained using Eqs (14 and 15) and taking into account the harmonic range from $\ell$ = −10 to $\ell$ = +10 of the OAM_−11_ mode generated in Fiber 1 at 835 nm wavelength. The OAM spectrum indicates a very high (99%) $\ell$ = −1 modal purity as expected, with residual peaks located at ±6 charges about the main harmonic ostensibly related to the six-fold symmetry of the photonic crystal structure. To verify that the high OAM mode purity is broadband, we computed in Fig. 11(b) the OAM_±11_ modes purities inside both optimized fibers within the range of simulated wavelengths from 600 to 1200 nm. One observes that the modal purity in Fiber 1 increases with the wavelength, while it decreases for Fiber 2. We attribute this behavior to the fact that Fiber 1 has a larger hole size (0.442 *μm*) compared to Fiber 2 (0.1 *μm*) such that modal confinement decreases with the wavelength in Fiber 2, whilst slightly improves in Fiber 1 for larger wavelengths. A similar relationship with the modal confinement was also observed for a different type of OAM fiber^26^.
